# Secretory activity of the coronary artery endothelial cells in conditions of the peritoneal dialysis

**DOI:** 10.1080/0886022X.2021.2023024

**Published:** 2022-02-17

**Authors:** Monika Misian, Ewa Baum, Andrzej Bręborowicz

**Affiliations:** Department of Pathophysiology, Poznan University of Medical Sciences, Poznań, Poland

**Keywords:** Peritoneal dialysis, coronary endothelium, inflammation, Sulodexide

## Abstract

**Introduction:**

Endothelial dysfunction is frequent in patients treated with peritoneal dialysis and may lead to cardiac complications. We evaluated the effect of effluent dialysates and serum on the function of coronary artery endothelial cells (CAEC).

**Methods:**

Human CAEC in *in vitro* culture were exposed to serum and dialysates from 24 patients treated with continuous ambulatory peritoneal dialysis (CAPD) and secretion of interleukin-6 (IL6), von Willebrand factor (vWF), tissue plasminogen activator (t-PA) and plasminogen activator inhibitor-1 (PAI-1) were measured. Modulation of the secretory activity of CAEC by Sulodexide, mixture of glycosaminoglycans: heparin sulfate and dermatan sulfate, was studied.

**Results:**

Serum from CAPD patients stimulated synthesis of IL6 (+93%), vWF (+18%), and PAI-1 (+20%) and did not change t-PA secretion in CAEC. Dialysates stimulated secretion of IL6 (+89%), vWF (+29%), and PAI-1 (+31%) and did not change t-PA synthesis. Dialysates collected in 12 patients after 6 months more strongly stimulated synthesis of IL6 (+37%) and PAI-1 (+7%). Sulodexide suppressed the secretory activity of CAEC stimulated by the studied sera: IL6 (–38%), vWF (–19%), t-PA (–13%), and PAI-1 (–12%).

**Conclusions:**

Serum and the dialysate from CAPD patients induce inflammatory and prothrombotic reaction in coronary arterial endothelial cells. The general pattern of the observed effects for serum and dialysates was similar but the intensity of the effects was not identical. Sulodexide reduced these effects.

## Introduction

Cardiorenal syndrome is a frequent cause of death in a group of patients with end-stage renal failure. Uremia causes dysfunction of the endothelium which is an important factor predisposing patients to the development of cardiorenal syndrome [1]. Renal replacement therapy removes molecules that are toxic toward the endothelial cells but at the same time initiates the development of their inflammatory phenotype [2,3]. One can assume that hemodialysis is more damaging than peritoneal dialysis, to the endothelial cells, because of the direct contact of blood with the dialysis membrane, which may induce intravascular inflammation. Hemodialysis performed with a cellulosic cuprophane membrane, contrary to a synthetic polysulfone membrane, more strongly impaired the endothelium-dependent flow-mediated dilation of the brachial artery [4]. In renal patients treated with hemodialysis, higher than in patients treated with peritoneal dialysis, levels of CD14+ and CD16+ monocytes and apoptotic endothelial microparticles were found [5]. On the other hand, in children treated with hemodialysis, stronger destruction of the endothelium was observed than during treatment with peritoneal dialysis [6]. However, both in patients treated with hemodialysis and peritoneal dialysis, significant damage to the endothelial glycocalyx was observed, which may disturb the function of the endothelium [7]. Endothelial dysfunction strongly correlates with cardiovascular complications in peritoneal dialysis patients [8]. In patients treated with peritoneal dialysis, dysfunction of the endothelium is also linked with the loss of residual renal function [9].

The peritoneal dialysis procedure induces intraperitoneal inflammation caused by the infusion of the bioincompatible dialysis fluids into the peritoneal cavity. Infusion of any solution into the peritoneal cavity is an unphysiological procedure *per se*, resulting in the induction of an inflammatory reaction. Second, some components of the peritoneal dialysis fluid are cytotoxic. Glucose degradation products (GDP) present in the dialysis fluid are cytotoxic toward mesothelial [10] and endothelial cells [11]. Park et al. reported that neutralization of the dialysate pH and minimization of the dialysate GDP significantly reduce the systemic level of the inflammatory markers and endothelial dysfunction in patients treated with chronic peritoneal dialysis [12]. However, in another study in continuous ambulatory peritoneal dialysis (CAPD) patients treated with physioneal, nutrineal, and extraneal (low GDP fluids), significantly higher serum levels of von Willebrand factor (vWF) and CRP were observed than in patients treated with the standard dialysis fluid containing a high level of GDP [13]. These observations suggest that peritoneal dialysis fluids with low levels of the potentially cytotoxic/injurious factors toward the mesothelial and endothelial cells are not the final recipe for the biocompatible procedure of that treatment. One can assume that the peritoneal dialysis induced intraperitoneal inflammation, independently of the dialysis solution used for treatment, always affects the intravascular space and the endothelial cells. We should know the characteristics and intensity of that reaction and how it affects various parts of the vascular system, especially the coronary blood vessels. Previously, we found that uremic serum collected from hemodialysis patients induces an inflammatory reaction in arterial endothelial cells and a prothrombotic effect in venous endothelial cells [14] .

We present results from a study in which the effects of overnight peritoneal dialysate effluents from CAPD patients dialyzed with high GDP fluid Dianeal 1.5% and their sera on the functional properties of coronary arterial endothelial cells were studied. Additionally, we show how these effects can be modulated by the drug Sulodexide, which is a mixture of natural glycosaminoglycans. Sulodexide has an anti-inflammatory and antithrombotic effect in venous diseases [15]. However, there are no data describing its effect in coronary artery endothelial cells (CAEC).

## Materials and methods

The study was done in a group of 24 CAPD patients (11 females and 13 males). In the studied group, diabetes mellitus (*n* = 8), hypertension (*n* = 8), glomerulonephritis (*n* = 6), amyloidosis (*n* = 1), and Alport syndrome (*n* = 1) were the causes of the end stage renal failure. Detailed data describing the studied population are shown in Table 1. The Bioethical Committee of the Poznan University of Medical Sciences approved the protocol of the study (decision 97/2019). All patients participating in the study were informed about the project and gave written consent to participate in the project. The study was performed according to rules of the Declaration of Helsinki.

**Table 1.** Clinical parameters of the studied population, presented as mean value ± SD.

Parameter	Mean value ± SD
Age (years)	51.9 ± 9.5
Time on dialysis (months)	25.7 ± 8.6
eGFR (mL/min/1.73 m^2^)	9.2 ± 6.5
Diuresis (mL/24 h)	1433 ± 437
Serum creatinine (mg/dL)	7.7 ± 3.3
Serum urea (mg/dL)	112.6 ± 30.9
Serum total protein (g/dL)	6.7 ± 0.5
Serum albumin (g/dL)	3.9 ± 0.4
Serum PTH (pg/mL)	363.8 ± 274.3
PET D/P creatinine	0.60 ± 0.13

Peritoneal dialysates and serum samples were collected from 24 patients after a 12 h overnight exchange performed with Dianeal 1.5% (Baxter, Deerfield, IL). No patients were diagnosed with peritonitis within three months prior to the dialysate collection. In 12 patients, dialysates were collected twice at six-month intervals. During that six-month intervals, no episodes of peritonitis or any systemic inflammatory disorders were observed. Also the general health status was not changed significantly during that period of time. After the dialysate drainage, it was centrifuged (200×*g*; 10 min), and the supernatant was frozen at −86 °C for further analysis.

### *In vitro* culture of endothelial cells

Experiments were performed on primary cultures of human CAEC purchased from Cell Applications, Inc. (San Diego, CA). The human endothelial cells growth medium provided by the producer of the cells was used for cell culture. Cells were grown to monolayers in 75 cm^2^ culture flasks, then harvested with trypsin 0.05%–EDTA 0.02% solution and seeded into 48 wells culture plates. Experiments were performed on the endothelial monolayers.

#### Effect of the serum and effluent dialysates on the secretory activity of endothelial cells

The dialysates and serum samples effects on the endothelial cells secretory activity were studied in separate experiments. Mean values of the inflammatory parameters of the patient's serum and dialysate samples used in the study are shown in Table 2. We found a correlation between the dialysate and serum IL6 concentrations (*r* = 0.478, *p*<.02)

**Table 2.** Concentration of the studied solutes in the dialysates and serum from CAPD patients (*n* = 24).

	Serum	Dialysate
IL-6 (pg/mL)	22.5 ± 9.7	57.8 ± 29.1, *p*<.001
t-PA (pg/mL)	1341 ± 726	954 ± 357, *p*<.05
PAI-1 (pg/mL)	35.752 ± 17.674	3355 ± 1868, *p*<.001
vWF (ng/mL)	342 ± 93	195 ± 64, *p*<.001

Statistical significance between the studied parameters in serum and dialysate is shown.

Serum samples from the peritoneal dialysis patients were added to the culture medium (20%). In the control group, serum from healthy, non-uremic donors (*n* = 12) was used at the same concentration (20%). Mean age of the healthy donors was 48.3 ± 9.8 years and in no person systemic disease was diagnosed or any therapy was used. Monolayers of CAEC in 48 wells plates were exposed for 24 h to the studied serum samples. Afterwards, the supernatant was removed from the wells and replaced with the standard culture medium for evaluation during the following 24 h of the incubation secretory activity of the cells. We found that such treatment did not induce any morphological changes in the endothelial cells and did not reduce viability when tested with the MTT test: 0.287 ± 0.054 in control medium and 0.267 ± 0.089 in medium with 20% serum (Sigma Aldrich, Gillingham, UK).

Effluent dialysates were mixed with the culture medium (1:1 v/v) and added to cells monolayers of CAEC in the 48 wells plates. In the control group, cells were exposed to the plain medium. Such treatment did not cause any damage to the endothelial cells measured with the MTT test: 0.296 ± 0.034 in control medium and 0.286  ±  0.066 in medium mixed with the dialysate (1:1 v/v). After 24 h of incubation, medium in all wells was replaced with the standard culture medium for evaluation of the cells secretory activity during the following 24 h. In both experiments, the medium was collected from all wells at the end of the 24 h incubation, spun down (200 g; 10 min) and frozen at −86 °C for further analysis. Cells were harvested with trypsin 0.05%–EDTA 0.02% solution (Sigma-Aldrich, St. Louis, MO) and counted in a hemocytometer. In the supernatants, concentrations of the following molecules: interleukin-6 (IL6), tissue plasminogen activator (t-PA), plasminogen activator inhibitor-1 (PAI-1), and vWF were measured with standard ELISA kits (R&D Systems, Minneapolis, MN). Secretion of the studied molecules from the endothelial cells was expressed per number of cells.

#### Effect of Sulodexide on the serum induced secretory activity of endothelial cells

In the separate set of experiments, we evaluated the effect of Sulodexide (0.5 LRU/mL) on the uremic serum-induced changes in the secretory activity of the endothelial cells. The concentration of Sulodexide used in the study reflected the level of that drug after its oral application [16]. The addition of Sulodexide to the medium did not reduce the viability of the cells measured with the MTT test: 0.276  ±  0.042 in control and 0.291  ±  0.067 in the presence of Sulodexide. Monolayers of CAEC in 48-wells plates were exposed for 24 h to the serum samples obtained from 24 patients and added to the culture medium (20%) ± Sulodexide 0.5 LRU/mL. At the end of the incubation, supernatants were removed from all wells and replaced with the culture medium to measure the secretory activity of the endothelial cells as described above.

### Statistical analysis

Results are presented as mean  ±  SD. Statistical analysis was performed with the Mann–Whitney or Wilcoxon’s test. Correlation between the studied groups was measured with the Spearman test. A *p* value less than .05 was considered statistically significant.

## Results

**Figure 1.**
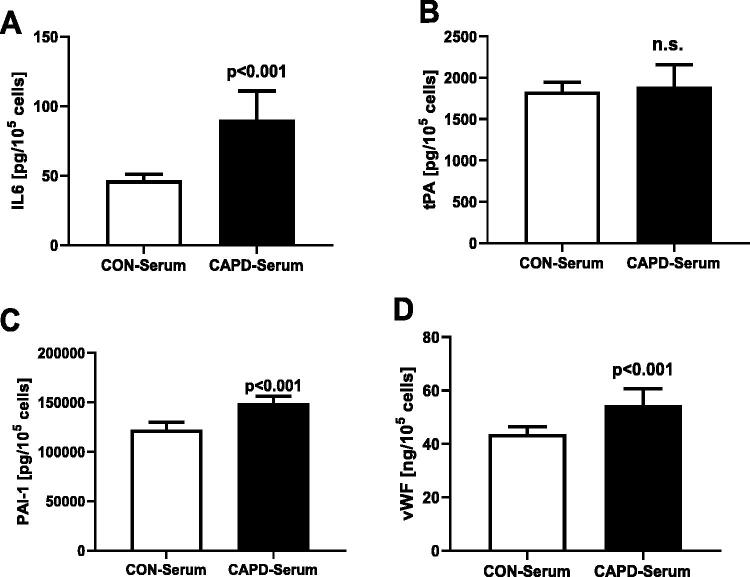
Effect of the control serum (CON) and serum from patients treated with continuous ambulatory peritoneal dialysis (CAPD) on secretion of IL6 (A), t-PA (B), PAI-1 (C), and vWF (D) in the human coronary arterial endothelial cells.

Serum samples collected from the peritoneal dialysis patients modified the secretory activity of the endothelial cells, as compared to the control serum. Synthesis of IL6, PAI-1, and vWF was increased by 93%, *p* < .001, 20%, *p* < .001 and by 18%, *p* < .001, respectively. No change in the synthesis of t-PA was observed (Figure 1). The ratio of t-PA/PAI-1 was lower in cells exposed to CAPD serum: 12.8 × 10^−3^ ± 1.9 × 10^−3^ than in the control group: 15.0 × 10^−3^ ± 1.2 × 10^−3^ (*p* < .001).

**Figure 2.**
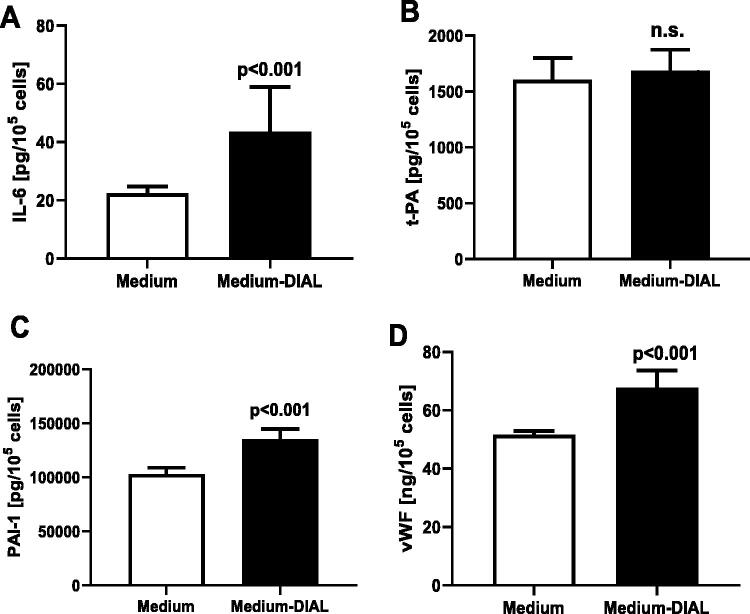
Effect of the culture medium (medium) and dialysates (medium-DIAL) from patients treated with the continuous ambulatory peritoneal dialysis on secretion of IL6 (A), t-PA (B), PAI-1 (C), and vWF (D) in the human coronary arterial endothelial cells.

Dialysates, analogically to CAPD serum, stimulated synthesis of IL6, PAI-1, and vWF, by 89%, *p* < .001, 31%, *p* < .001, and by 29%, *p* < .001, respectively, and no change in t-PA synthesis was observed (Figure 2). The ratio of t-PA/PAI-1 was lower in cells exposed to CAPD dialysate: 12.5 × 10^−3^ ± 1.7 × 10^−3^ than in the control group: 15.7 × 10^–3^ ± 2.3 × 10^−3^ (*p* < .001). We found a correlation between the CAPD dialysate and serum effect on the synthesis of IL6 in the endothelial cells (*r* = 0.614, *p* < .002).

**Figure 3.**
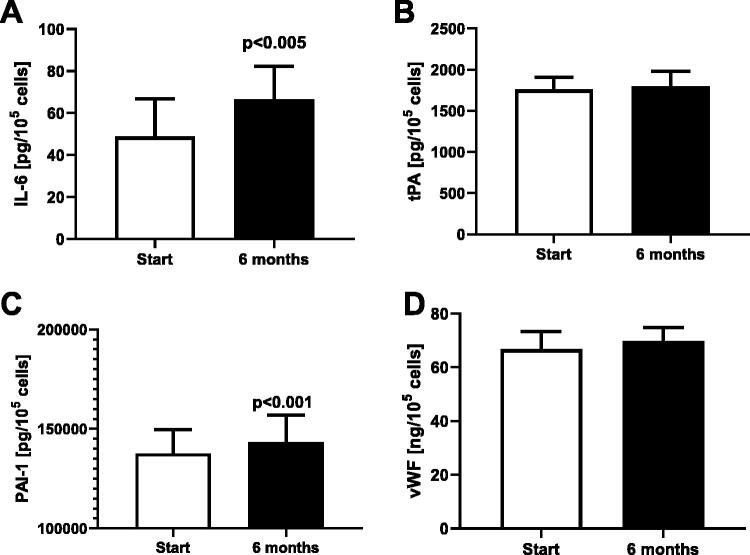
Effect of the dialysates collected from patients treated with the continuous ambulatory peritoneal dialysis at the beginning of the study (start) and after 6 months (6 months) on secretion of IL6 (A), t-PA (B), PAI-1 (C), and vWF1 (D) in the human coronary arterial endothelial cells.

Dialysates collected in 12 patients after six months of therapy had a higher level of IL6, as compared to the beginning of the study: 78.0  ±  37.9 pg/mL vs. 57.8  ±  29.1 pg/mL, *p* < .01. No differences in other parameters were detected. Dialysates collected after six months of the therapy stronger stimulated synthesis of IL6 and PAI-1 in the endothelial cells, as compared to the beginning of the study: +37%, *p* < .005 and +7%, *p* < .001, respectively. No change in the synthesis of t-PA and vWF was observed (Figure 3). After 6 months of therapy t-PA/PAI-1 ratio was reduced from 12.9 × 10^−3^ ± 1.6 × 10^−3^ to 12.3 × 10^−3^ ± 1.4 × 10^−3^, *p* < .005.

**Figure 4.**
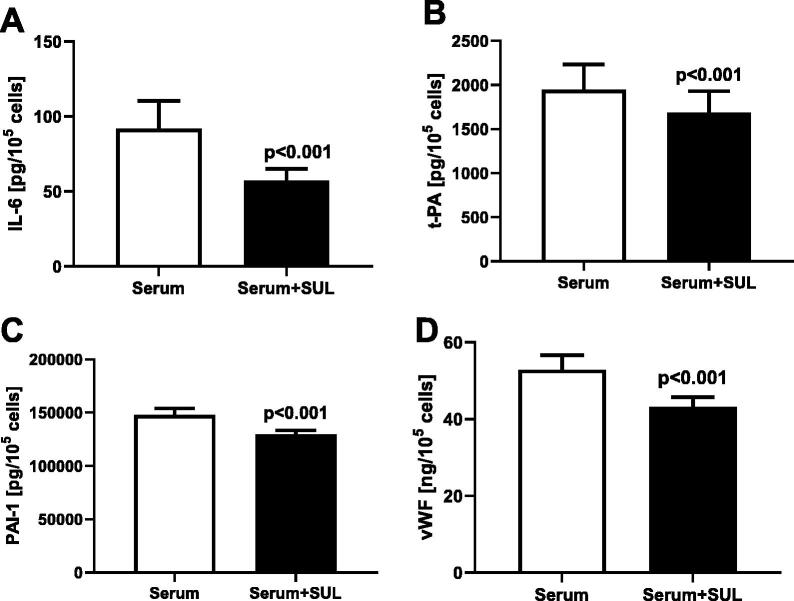
Effect of the studied serum (serum) supplemented with Sulodexide 0.5. LRU/mL (serum + SUL) on secretion of IL6 (A), t-PA (B), PAI-1 (C), and vWF (D) in the human coronary arterial endothelial cells.

Sulodexide suppressed the secretory activity of the endothelial cells during their exposure to the CAPD sera. Synthesis of IL6 was reduced by 38%, *p* < .001, t-PA by 13%, *p* < .001, PAI-1 by 12%, *p* < .001 and vWF by 19%, *p* < .001 (Figure 4). No change in the t-PA/PAI-1 ratio was detected.

## Discussion

Peritoneal dialysis induces intraperitoneal inflammation, which is caused by repeatedly infused bioincompatible dialysis fluids into the abdominal space and depends, as we found in the present study, on the individual reaction of each patient. We studied effluents after 12 h of the dwell, but inflammatory mediators are also present in the dialysates collected after shorter exchanges which make intraperitoneal inflammation constant process in peritoneal dialysis patients. The wide range of IL6 dialysate levels observed in our study (20.3–118.7 pg/mL) confirms that the patients' reaction to the infused dialysis fluids significantly determines the intensity of the intraperitoneal inflammation. Higher levels of IL6 in the dialysates as compared to serum suggest that the intraperitoneal inflammatory reaction translates into an intravascular one. That statement is confirmed by the correlation observed in our study between the dialysate and serum IL6 levels (*r* = 0.478, *p* < .02). Both the patients' serum and dialysates stimulated the synthesis of IL6 in coronary endothelial cells. These results suggest that the peritoneal dialysate may partially indirectly affect the function of the endothelial cells through the induction of intravascular inflammation and changes in the plasma properties. The systemic inflammatory reaction leads to endothelial dysfunction in peritoneal dialysis patients [17]. There is a strong correlation between endothelial dysfunction and cardiovascular complications in peritoneal dialysis patients [8]. The relationship between increased IL6 levels and the progression of atherosclerosis is well known [18]. The correlation observed in our study between the dialysates and sera effects of the endothelial secretion of IL6 suggests that the dialysate plays a significant role in the induction of the intravascular inflammatory reaction. Additionally, we found that the proinflammatory effect of the dialysates, as reflected by the stimulation of IL6 synthesis in the endothelial cells, is increased with the time of the renal replacement treatment. These findings confirm clinical observations showing, proportional with the time of therapy, an increase of intraperitoneal and systemic inflammation [19]. That means that the atherosclerotic effect of the peritoneal dialysate increases with time.

Both sera and dialysates stimulated the synthesis of vWF and PAI-1 in the endothelial cells, which means that not only was the procoagulant activity of these cells enhanced but at the same time their fibrinolytic potential was reduced. Tomura et al. found higher blood levels of PAI-1 in CAPD patients as compared to patients treated with hemodialysis or healthy controls [20]. The reduced blood fibrinolytic activity may predispose patients to the development of atherosclerosis [21]. We found that with the time of treatment peritoneal dialysate more strongly stimulates synthesis of PAI-1, which may further impair the fibrinolytic activity of the coronary endothelium. Results from clinical studies show that increased blood vWF level predicts the risk of vascular disorders and cardiac mortality in CAPD patients [22,23]. Results from our study suggest that at least part of the adverse effects of serum from CAPD patients on the endothelium are secondary to the dialysates properties. Therefore, prevention of vascular disorders in patients on chronic peritoneal dialysis should be focused on the reduction of intraperitoneal inflammation and direct protection of the endothelial cells.

The application of dialysis fluids with low osmolality and/or low concentration of GDP does not always result in a reduction of intraperitoneal and systemic inflammation [12,13]. We found previously that supplementation of the dialysis fluids with hyaluronan suppresses dialysate-induced intraperitoneal inflammation [24]. In the present study, Sulodexide, which is a mixture of natural glycosaminoglycans: heparin sulfate and dermatan sulfate, suppressed the stimulatory effect of the studied sera on the secretory activity of coronary endothelial cells. That means that not only the inflammatory but also their prothrombotic action was reduced, which is a positive effect. Previously, we found that Sulodexide reduced the proinflammatory effect of serum from patients with peripheral venous or arterial diseases on venous and arterial endothelial cells [25,26]. Sulodexide also inhibits intraperitoneal inflammation and reduces the dialysate proinflammatory and profibrotic effects [27,28]. These data suggest that Sulodexide has the potential for suppression of intraperitoneal and systemic inflammation in CAPD patients. Additionally, the use of Sulodexide may result in a decreased risk of thrombotic disorders, which are present in patients treated with chronic peritoneal dialysis [29,30]. A decrease in the blood vWF may reduce the risk of cardiovascular disorders [22,23]. Previously, we found that Sulodexide reduced vWF secretion from human endothelial venous cells exposed to serum from uremic patients treated with hemodialysis [31]. On the other hand, Kim et al. found that treatment with Sulodexide in peritoneal dialysis patients decreased plasma D-dimers as an index of coagulation, but there was no significant change in blood vWF level [32].

In conclusion, we found that peritoneal dialysate induces directly and indirectly via its effect on the serum properties, inflammatory and procoagulant reaction in coronary endothelial cells, which may translate into a higher risk of various pathologies such as atherosclerosis or thrombotic disorders. The intensity of such effects increases with the time of the renal replacement therapy. Sulodexide can partially prevent these effects.
